# The effects of a thermogenic supplement on resting metabolic rate in healthy males: preliminary results

**DOI:** 10.1186/1550-2783-11-S1-P47

**Published:** 2014-12-01

**Authors:** Ryan Colquhoun, Bill Campbell, Gina Zito, Nic Martinez, Laura Buchanan, Matt Lehn, Mallory Johnson, Courtney St Louis, Yasmin Smith, Brad Cloer, Allison Pingel

**Affiliations:** 1 University of South Florida, Tampa, Florida, USA

## Background

Males looking to improve their body composition may ingest caffeine-containing supplements for the purposes of elevating resting metabolic rate. The purpose of this study was to examine the effects of a commercially available dietary supplement (containing ingredients that promote thermogenesis) on resting metabolic rate (RMR) in a randomized, double-blind, placebo-controlled cross-over study.

## Methods

8 male participants (30.1 ± 10.0 years; 181.1 ± 9.0 cm; 84.8 ± 13.0 kg) volunteered to participate in this investigation. Each participant underwent two different testing sessions separated by approximately 7 days. On their first visit, participants arrived to the laboratory after an overnight fast and underwent a baseline RMR. Following this, each participant ingested a caffeine-containing dietary supplement (Arnold Iron CutsTM) or a placebo and repeated the RMR assessments at 30, 60, 90, 120, and 180 minutes post-ingestion. The placebo was void of active ingredients known to elevate RMR. Approximately 1-week later, the alternative supplement was ingested and the assessments were repeated in the exact same manner. Data were analyzed via a 2-factor [2x4] within-subjects repeated measures analysis of variance (ANOVA) using SPSS version 22.0. Post-hoc tests were analyzed via paired samples t-tests. The criterion for significance was set at p ≤ 0.05. Consent to publish the results was obtained from all participants.

## Results

The repeated measures ANOVA revealed a significant effect for time relative to the raw RMR data. Post-hoc analyses revealed that the dietary supplement treatment demonstrated significant elevations in RMR (kilocalories/day) at 30-minutes, 60-minutes, and 180-minutes post-ingestion (p ≤ 0.05) and demonstrated statistical trends at 90 and 120-minutes post-ingestion (p ≤ 0.10). There were no significant elevations (or statistical trends) at any time point in the placebo treatment. Table 1 demonstrates the raw data (mean ± SD) and the percentage increases in RMR for each time point for both supplement groups.

**Table 1:** 

	Baseline	30-minute	60-minute	90-minute	120-minute	180-minute
Arnold Iron Cuts^TM^	1,880 ± 202	2,004 ± 133 (6.6.%)^*^	2,033 ± 92 (8.1%)^*^	2,048 ± 103 (8.9%)^#^	2,013 ± 75 (7.1%)^#^	2,067 ± 116 (9.9%)^*^

Placebo	1,908 ± 236	1,995 ± 301 (4.6.%)	1,971 ± 253 (3.3%)	1,969 ± 316 (3.2%)	1,984 ± 241 (4.0%)	1,919 ± 105 (0.5%)

^#^ - Post-hoc statistical trend compared to baseline values (p ≤ 0.10)

^*^ - Post-hoc statistical difference compared to baseline values (p ≤ 0.05)

## Conclusion

The caffeine-containing dietary supplement treatment exerted greater elevations in RMR values as compared to the placebo treatment. Taken on a daily basis, Arnold Iron CutsTM may increase overall energy expenditure possibly leading to reductions in fat mass over time. Caloric expenditure either significantly increased or demonstrated statistical trends for improvement at each time point, whereas the placebo treatment experienced no change in energy expenditure.

